# Comparative efficacy and safety of alternatives to sodium valproate in the management of bipolar affective disorder in people of child-bearing age: a narrative review by the European Society of Clinical Pharmacy’s mental health specialist interest group

**DOI:** 10.1007/s11096-025-01919-x

**Published:** 2025-04-28

**Authors:** Ita Fitzgerald, Izgi Bayraktar, Birgit Eiden, Rosalind Gittins, Erica Magni, Marie Humbert-Claude, Lara-Turiya Molitschnig, Paula Darm, Anna Waksmundzka-Walczuk, Nikolaus Riesenhuber, Matej Stuhec, Ivana Tašková, Martina Hahn

**Affiliations:** 1 Pharmacy Department, St Patrick’s Mental Health Services, St Patrick’s University Hospital, Dublin 8, D08K7YW Ireland; 2 School of Pharmacy, University College Cork, Cork, Ireland; 3 Department of Clinical Pharmacy, Faculty of Pharmacy, Hacettepe University, Ankara, Turkey; 4 Pharmacy Department, Rheinhessen, Fachklinik Alzey, Alzey, Germany; 5 Aston School of Pharmacy, Aston University, Birmingham, UK; 6 Pharmacy Department, ASP IMMeS e PAT, Milan, Italy; 7 Pharmacy of the Eastern Vaud Hospitals, Route du Vieux Séquoia 20, 1847 Rennaz, Switzerland; 8 Pharmacy Department, Hospital of the Elisabethians Graz, Graz, Austria; 9 Department of Psychiatry, Psychosomatics and Psychotherapy, University Hospital Frankfurt-Goethe University, Frankfurt, Germany; 10 District Health Care Institute, Starachowice Hospital, Starachowice, Poland; 11 Pharmacy Department, Medical University of Vienna, Vienna, Austria; 12 Faculty of Medicine, University of Maribor, Maribor, Slovenia; 13 Department of Clinical Pharmacy, Ormoz Psychiatric Hospital, Ormoz, Slovenia; 14 Psychiatric Hospital Bohnice, Prague, Czech Republic; 15 Department of Applied Pharmacy, Faculty of Pharmacy, Masaryk University, Brno, Czech Republic; 16 Department of Mental Health, Varisano Hospital, Frankfurt, Germany; 17 Faculty of Pharmacy, Institute for Pharmacology and Clinical Pharmacy, Philipps-University Marburg, Marburg, Germany

**Keywords:** Bipolar affective disorder, Bipolar disorder, Mood stabilisers, Narrative review, Pregnancy, Sodium valproate, Teratogenicity

## Abstract

**Background:**

The European Medicines Agency has recommended a series of restrictions on the use of sodium valproate (valproate) following research linking its exposure in utero to adverse congenital and neurodevelopmental effects in offspring. Recent research has highlighted a potential increased risk of neurodevelopmental disorders in children born to males taking valproate prior to conception. Clinicians and patients require guidance regarding suitable alternatives.

**Aim:**

To provide an overview of suitable alternatives to valproate in the management of bipolar disorder.

**Method:**

A narrative review was conducted. Only medications with an established evidence base in managing different phases of bipolar disorder and endorsed within clinical practice guidelines were considered. Eligible guidelines included those (i) where recommendations were informed by a formal guideline development process and (ii) published in English within the last 15 years. REPROTOX® was chosen as the primary information source regarding reproductive safety of alternative medications.

**Results:**

Of all second-generation antipsychotics, quetiapine should be considered a first-line alternative to valproate. Lithium has been associated with an increased risk of cardiac malformations, especially Ebstein anomaly, following in utero exposure. However, given its robust efficacy as an antimanic agent and the absolute risk of cardiac abnormalities being low, it’s use can still be considered in individuals of child-bearing potential with appropriate monitoring. Carbamazepine treatment should be avoided due to concerns for teratogenicity. Although considered safe in pregnancy, lamotrigine is largely effective at preventing relapse of bipolar depression. Thus, lamotrigine offers limited clinical utility as an alternative to valproate.

**Conclusion:**

Specific recommendations are made regarding alternatives to valproate in managing bipolar disorder.

## Impact statements

- The European Medicines Agency has recommended a series of restrictions on the use of sodium valproate in both males and females of child-bearing age. This narrative review provides an overview of alternative mood stabilising medications for patients and clinicians to consider in managing bipolar disorder in people planning a pregnancy.
- Of all second-generation antipsychotics, quetiapine should be considered as a first-line alternative to valproate. Aripiprazole and olanzapine can be considered as alternative antipsychotics where quetiapine is not clinically suitable.
- Although lithium exposure *in utero* has been consistently demonstrated to increase risk of primarily cardiac abnormalities, absolute risk estimates available are low. Thus, given lithium’s robust efficacy as an antimanic agent, it should be considered as one appropriate alternative to valproate.
- Based on currently available evidence, carbamazepine should be avoided due to evidence of teratogenicity, including neural tube defects and craniofacial abnormalities.
- Lamotrigine has demonstrated efficacy in the management of depressive episodes of bipolar disorder, and therefore, offers limited clinical utility as an alternative to valproate.

## Introduction

In 2018, the European Medicines Agency (EMA) recommended new restrictions on the use of sodium valproate (from here referred to as ‘valproate’) including a requirement that females of child-bearing age prescribed valproate for any indication be initiated on a Pregnancy Prevention Programme [1]. Such measures were introduced to reduce in utero exposure among children born to mothers prescribed valproate due to a high risk of subsequent congenital malformations and neurodevelopmental adverse effects [2]. Specifically, in utero exposure to valproate has been associated with an approximately 11% increased risk of congenital disorders and an up to 30–40% increased risk of neurodevelopmental disorders [3]. Empirical evidence supporting valproate use in bipolar disorder is most established in the management of acute mania and prevention of future episodes [4, 5]. Use of valproate in managing or preventing episodes of bipolar depression is less well established [6]. In the management of mixed affective disorders, valproate is recommended as a potential treatment within some clinical practice guidelines (CPGs) [7, 8]. Among those with rapid cycling bipolar disorder, valproate is also recommended as an appropriate maintenance mood stabilising medication [6].

Given its wide-ranging indications, EMA restrictions on the prescribing of valproate impacts a substantial number of individuals living with bipolar disorder. This includes those already established on valproate treatment and those requiring effective management of bipolar disorder predominated by manic episodes [6–8]. To support timely access to effective care, clinicians and patients require ease of access to comprehensive resources outlining suitable alternatives to valproate. This includes alternatives supported by a similar empirical evidence base in managing the range of presentation where valproate was previously indicated, and those are considered safer among individuals of child-bearing age or those actively planning a pregnancy. This narrative review provides an overview of potential alternatives to valproate in the acute management and prevention of episodes of (i) bipolar depression, (ii) mania, (iii) mixed mood, and (iv) rapid cycling in the context of bipolar disorder.

Of note, ‘valproate’ refers to three available formulations containing the medication: sodium valproate, valproic acid and semi-sodium valproate. Both semi-sodium and sodium valproate are metabolised to valproic acid (also known as valproate), which is the pharmacologically active component [9].

### Aim

To provide an overview of potentially suitable alternatives to valproate in the management of bipolar disorder among individuals of child-bearing age, particularly those planning a pregnancy.

## Method

Given the focus on providing suggested alternatives to valproate, only medications with an established evidence base, and thus, endorsed within CPGs in the management of (i) bipolar depression, (ii) mania, (iii) mixed mood episodes and (iv) rapid cycling were considered. This includes in the treatment of acute episodes and relapse prevention. When identifying CPGs for inclusion, guidelines considered eligible included those that were: (i) informed by a formal guideline development process, including a systematic review of primary research, (ii) published in English and within the last 15 years. Authors also considered the relevance of identified guidelines in informing practice at a European and international level. Guidelines were searched for and screened by team members using databases including PubMed and Google Scholar. Three guidelines were subsequently included:

1. The National Institute for Health and Care Excellence (NICE) Bipolar disorder: assessment and management (2014) [9]
2. Canadian Network for Mood and Anxiety Treatments (CANMAT) and International Society for Bipolar Disorders (ISBD) guidelines for:
  1. The management of patients with bipolar disorder (2018) [6]
  2. The management of patients with bipolar disorder with mixed presentations (2021) [7]
3. The World Federation of Societies of Biological Psychiatry (WFSBP) Guidelines for the Biological Treatment of Bipolar Disorders:
  1. Acute and long-term treatment of mixed states in bipolar disorder (2017) [8]
  2. Maintenance treatment of bipolar disorders (2013) [10]
  3. Acute bipolar depression (2010) [11]
  4. Treatment of acute mania (2009) [12]

Due to their questionable efficacy in managing bipolar depression and concerns regarding risks, use of classic antidepressants is not considered here.

Following review of guideline recommendations, individual medications reviewed for their safety during pregnancy included:

1. Lithium
2. Lamotrigine
3. Carbamazepine
4. Second-generation antipsychotics (aripiprazole, haloperidol, olanzapine, risperidone, quetiapine)

Information regarding teratogenicity of each medication was identified via use of the REPROTOX® database [13]. REPROTOX® contains summaries on the effects of medications on fertility, pregnancy, and neonatal development. REPROTOX® was chosen as the primary information source regarding risks associated with in utero exposure of mood stabilising medications as it is updated regularly (at least yearly) and is informed by systematic literature reviews [13]. Primary risk considerations are listed here as the basis of informing alternative mood stabilising medications. However, the manufacturer’s summary of product characteristics and other sources of evidence-based, contemporaneous information should always be referred to in addition by prescribers.

## Results

This section outlines key points for clinicians when considering suitable alternatives to valproate among individuals of child-bearing potential, with a focus on safety following *in utero* exposure. Medication efficacy within the context of managing differing aspects of bipolar disorder is discussed, although a more detailed overview, including the level of supporting evidence, is contained within Tables 1, 2, 3, 4, 5. Of note, the most recent guidelines considered are those produced by CANMAT [6, 7]. Specific advice for clinicians in the case of each medication as to whether it can be considered as a suitable alternative to valproate is listed as the end of each corresponding paragraph.

**Table 1:** Clinical practice guideline recommendations for treating acute mania

Medication/recommendation addressing use within guideline + level of supporting evidence (where available)	CANMAT [6, 7]	NICE [9]	WFSBP [8–12]
Aripiprazole	1st line (level 1 evidence)	No specific recommendations addressing use	Evidence A (grade 1)
Carbamazepine	2nd line (level 1 evidence)	No specific recommendations addressing use	Evidence A (grade 2)
Haloperidol	2nd line (level 1 evidence) - only combination therapies	1st line	Evidence A (grade 2)
Lithium	1st line (level 1 evidence)	3rd line (add-on)	Evidence A (grade 2)
Lamotrigine	Not recommended (level 1 negative)	Not recommended	Evidence E (not recommended)
Quetiapine	1st line (level 1 evidence)	1st line	Evidence A (grade 2)
Olanzapine	2nd line (level 1 evidence)	1st line	Evidence A (grade 2)
Risperidone	1st line (level 1 evidence)	1st line	Evidence A (grade 1)

**Table 2:** Clinical practice guideline recommendations for treating acute bipolar depression

Medication/recommendation addressing use within guideline + level of supporting evidence (where available)	CANMAT [6, 7]	NICE [9]	WFSBP [8–12]
Aripiprazole	3rd line adjunct treatment option	No specific recommendations addressing use	Evidence E (no recommendation grade provided)
Carbamazepine	3rd line (level 2 evidence)	No specific recommendations addressing use	Evidence D (grade 5)
Haloperidol	No specific recommendations addressing use	No specific recommendations addressing use	No specific recommendations provided addressing use
Lithium	1st line (level 2 evidence)	If already on lithium increase dose and/or add additional agent	Evidence D (grade 5) If already on lithium, dose increase: Evidence B (grade 3), or use of lithium as combination treatment: Evidence level B-D (grade 3–5) depending on agent
Lamotrigine	1st line (level 2 evidence)	2nd Line	Evidence B (grade 3)
Quetiapine	1st line (level 1 evidence)	1st line	Evidence A (grade 1)
Olanzapine	2nd Line (level 1 evidence)	2nd Line (1st line if used in combination with fluoxetine)	Evidence B (grade 3)
Risperidone	No specific recommendations addressing use	No specific recommendations addressing use	No specific recommendations addressing use

**Table 3:** Clinical practice guideline recommendations for preventing relapse of manic episodes

Medication/recommendation addressing use within guideline + level of supporting evidence (where available)	CANMAT [6, 7]	NICE [9]	WFSBP [8–12]
Aripiprazole	1st line (level 2 evidence)	2nd line	Evidence A (grade 1)
Carbamazepine	2nd line (level 2 evidence)	No specific recommendations addressing use	Evidence F (grade 4)
Haloperidol	2nd line (level 4 evidence)	No specific recommendations addressing use	Not recommended
Lithium	1st line (level 1 evidence)	1st line	Evidence B (grade 1)
Lamotrigine	1st line (level 3 evidence)	No specific recommendation	Evidence D (no recommendation grade provided)
Quetiapine	1st line (level 1 evidence)	2nd line therapy	Evidence A (grade 1)
Olanzapine	2nd line (level 1 evidence)	2nd line therapy	Evidence A (grade 2)
Risperidone	2nd line (level 2 evidence)	2nd line	Evidence A (grade 2)

**Table 4:** Clinical practice guideline recommendations for preventing relapse of bipolar depression

Medication/recommendation addressing use within guideline + level of supporting evidence (where available)	CANMAT [6, 7]	NICE [9]	WFSBP [8–12]
Aripiprazole	For any episode: first line (level 2 evidence)	No specific recommendations addressing use	Evidence E (no recommendation grade provided)
Carbamazepine	2nd line (level 2 evidence)	No specific recommendations addressing use	Evidence F (grade 4)
Haloperidol	Not recommended (level 4 negative evidence)	No specific recommendations addressing use	Not recommended
Lithium	1st line (level 1 evidence)	1st line	Evidence B (grade 1)
Lamotrigine	1st line (level 1 evidence)	No specific recommendations	Evidence A (grade 1)
Quetiapine	1st line (level 1 evidence)	2nd line therapy	Evidence A (grade 1)
Olanzapine	2nd line (level 1 evidence)	2nd line therapy	Evidence B (grade 2)
Risperidone	2nd line Risperidone LAI (long-acting injection) 2nd line (level 4 evidence) Risperidone LAI (as an adjunctive treatment)	2nd line	Evidence E (no recommendation grade provided)

**Table 5:** Clinical practice guideline recommendations for treating mixed episodes

Medication/recommendation addressing use within guideline + level of supporting evidence (where available)	CANMAT [6, 7]	NICE [9]	WFSBP [8–12]
Aripiprazole	No specific recommendations addressing use	Not recommended	Evidence B in manic mixed states (grade 3), Evidence F in depressive mixed states (no recommendation grade provided)
Carbamazepine	No specific recommendations addressing use	Not recommended	Evidence C (grade 4 for manic mixed states when used as monotherapy, no recommendation grade provided for depressive mixed states)
Haloperidol	No specific recommendations addressing use	Not recommended	Evidence F (grade 4)
Lithium	No specific recommendations addressing use	3rd line (add-on)	Evidence F (not recommended)
Lamotrigine	No specific recommendation addressing use	No specific recommendation addressing use	Evidence F (not recommended)
Quetiapine	1st line (level 1 evidence)	1st line	Evidence E for manic mixed states (grade 3), Evidence F for depressive mixed states (grade 3 if used as combination therapy in depressive mixed states)
Olanzapine	- (only in combination with fluoxetine)	1st line	Evidence A for manic mixed states (grade 2), evidence C for depressive mixed states (grade 4)
Risperidone	No specific recommendations addressing use	1st line	Evidence C for manic mixed states (grade 4 if used as combination treatment), Evidence F for depressive mixed states (no recommendation grade assigned)

Of note, medications are listed in alphabetical order rather than order of recommended preference as an alternative to valproate.

### Aripiprazole

Aripiprazole is a third-generation antipsychotic with demonstrated efficacy in the managing acute mania and mixed episodes within bipolar disorder [14]. CANMAT guidelines recommend aripiprazole monotherapy as a first-line treatment in acute agitation. CANMAT also recommends aripiprazole as a first-line intervention in managing acute mania and second-line for the prophylaxis of both mania and depression. Within the 2023 update, aripiprazole was included as a third-line adjunct for the management of acute depressive episodes in bipolar 1 disorder and a first-line intervention in preventing any episode [6, 7]. NICE recommends aripiprazole as an alternative to lithium in the maintenance therapy of bipolar disorder in cases where lithium treatment is inappropriate [9]. WFSBP guidelines recommend aripiprazole in the management of acute mixed, manic and depressive episodes, and in the prevention of any episode of bipolar disorder [10–12].

Aripiprazole has not been shown to increase risk of major malformations within human studies [15]. Reports from teratology information services, birth registries and pregnancy registries have not shown an increase in the prevalence of malformations following aripiprazole exposure in utero. Additionally, compared with other antipsychotics, aripiprazole has not been associated with increased risk of gestational diabetes [15]. Given its efficacy in managing primarily manic episodes of bipolar disorder and its demonstrated safety in pregnancy, aripiprazole can be considered a safe alternative to valproate in the context of treating acute manic episodes and in preventing their relapse.

### Carbamazepine

Carbamazepine is a mood stabilising medication with an evidence base primarily in managing acute mania [16]. Whilst NICE make no recommendations regarding its use [9], CANMAT recommend carbamazepine as a second-line treatment within acute mania and in the prevention of any mood episode [10, 12]. Carbamazepine has been demonstrated as having teratogenic potential [17]. Its use during early pregnancy has been associated with neural tube defects and craniofacial abnormalities. In utero exposure has also been associated with developmental delays [17]. Considering this, and the availability of effective and safer alternatives carbamazepine should not be considered a suitable alternative to valproate among individuals of child-bearing potential.

### Haloperidol

Within NICE guidelines, haloperidol is recommended as a first-line intervention managing acute mania and hypomania [9]. CANMAT guidelines recommend haloperidol as a short-term treatment for agitation, or as a second-line treatment in acute mania [6]. WFSBP guidance recommends treatment with haloperidol in managing acute mania [12]. In the context of maintenance treatment, haloperidol is generally not recommended due to issues of tolerability and a lack of documented evidence in support of continued efficacy [6, 9, 12]. Haloperidol exposure in utero has not been associated with an increased risk of congenital abnormalities. There is, however, a specific concern regarding withdrawal effects in neonates following third trimester exposure [18]. Given the availability of alternative antipsychotics with a more robust evidence base in preventing manic relapse, haloperidol should be avoided as a substitute maintenance treatment among those established on valproate and requiring ongoing treatment.

### Lithium

Lithium is a mood stabilising medication with demonstrated efficacy in the treatment and prophylaxis of both manic, and to a lesser extent, depressive episodes of bipolar disorder [19]. CANMAT lists lithium among first-line options in the treatment and prophylaxis of mania and bipolar depression [6]. NICE recommend use of lithium after insufficient initial response to antipsychotic treatment within acute mania but recommend its use as first-line maintenance treatment [9].WFSBP guidelines recommend lithium in managing acute mania, as well as in preventing relapse of any mood episode [8–10, 12]. Lithium exposure in utero has been associated with an increased risk of primarily cardiac malformations, particularly Ebstein anomaly following first trimester maternal exposure, with associated risk estimates reported to be around 1 in 2,000 live births, a tenfold increase compared to the risk in non-lithium exposed live births [20]. Lithium exposure in utero during any trimester has also been associated with preterm birth, hypothesized to be attributable to lithium's propensity to cause subclinical or clinical hypothyroidism [20, 21].

Given its long-established evidence base in managing bipolar disorder, particularly in treating and preventing episodes of mania, lithium remains a suitable alternative to valproate, particularly among individuals where monotherapy with second-generation antipsychotics will likely be, or has been demonstrated to be, clinically insufficient to maintain mental health. Among those planning a pregnancy where second-generation antipsychotic treatment is appropriate, quetiapine treatment should likely be given priority over lithium. Changes in pharmacokinetic parameters associated with lithium use during pregnancy and the need for additional therapeutic drug monitoring add complexity to lithium use during pregnancy [20].

### Lamotrigine

Lamotrigine is a mood stabilising medication with an evidence base primarily in preventing episodes of bipolar depression. It is not recommended in treating acute manic episodes [6, 9] and has been demonstrated as less effective than lithium in preventing their reoccurrence [22]. Although recommended in treating acute bipolar depression within some guidelines [6, 9], the slow speed of titration required to mitigate risk of serious skin reactions, including Steven’s Johnson Syndrome [23], makes use of lamotrigine within acute presentations largely impractical. Documented effect sizes in treating acute bipolar depression are also comparatively smaller to other mood stabilisers, including quetiapine and lithium [24]. Within CANMAT and WFSBP guidelines, lamotrigine is recommended as monotherapy, or in combination with other mood stabilisers (particularly lithium), among those initiating or switching treatment during the maintenance phase of bipolar depression. [6, 10].

Lamotrigine exposure in utero is not thought to be associated with an increased risk of congenital malformations [25]. Although previously thought to increase risk of oral cleft palate (estimated 1% increase from background rate), this has not subsequently been confirmed [25]. Given its primary clinical utility being in preventing episodes of bipolar depression and alternatives with a more robust evidence base in preventing episodes of mania, lamotrigine should not be considered a suitable alternative to valproate.

### Olanzapine

Olanzapine is most effective in managing acute mania within bipolar disorder, where it is recommended as a first-line treatment by NICE and WFSBP [9, 12], and as a second-line treatment by CANMAT [6]. Due to a less robust evidence base within acute bipolar depression, olanzapine is considered within these contexts a second-line treatment by CANMAT [6], WFSBP [11], and NICE (although NICE recommend consideration as a first-line intervention if used in combination with fluoxetine) [9]. For the prevention of any future episodes, olanzapine is recommended a second-line treatment option within most guidelines [6, 9, 10].

Most studies have not identified an association between olanzapine exposure in utero and an increased risk of congenital malformations or neurodevelopment disorders [26]. Olanzapine is, however, associated with an increased risk of adverse maternal effects, particularly gestational diabetes [21]. Risk may depend on other factors with one population-based cohort study showing risk of gestational diabetes to vary significantly, adjusted odds ratio = 1.94 (95% CI 0.97–3.91) [27]. Given its demonstrated efficacy and safety, olanzapine can be considered as one suitable alternative to valproate. However, among those requiring maintenance treatment, particularly those actively planning pregnancy, quetiapine offers a more effective and safer alternative second-generation antipsychotic [16, 28].

### Quetiapine

Quetiapine has an established evidence base in the acute treatment and prevention of manic, depressive, and mixed episodes in bipolar disorder [6, 16, 24, 28]. Quetiapine also has an established evidence base in the treatment of both mixed episodes and rapid cycling BPAD [7]. Quetiapine is recommended within CANMAT guidelines as a first-line treatment in managing acute mania and bipolar depression and as a maintenance therapy for the prevention of any mood episode [6, 7]. Within WFSBP guidelines quetiapine is recommended as a second-line treatment for acute mania, but a first-line treatment for acute bipolar depression. WFSBP guidance also recommends quetiapine for preventing episodes of mania and bipolar depression [8, 10–12]. NICE recommend quetiapine for treatment of acute manic and depressive episodes in bipolar disorder and as a maintenance treatment following established response [9]. Quetiapine is also recommended for the treatment of mixed episodes and rapid cycling by CANMAT and NICE [6, 7, 9].

Quetiapine has not been demonstrated to significantly increase risk of congenital anomalies following in utero exposure [29]. Previous registry studies have reported quetiapine use during pregnancy to be associated with a higher risk of infants being larger than gestational age, adjusted risk ratio (aRR) = 1.32 (95% CI 1.06–1.63) [29]. Administration during pregnancy has also been associated with an increased risk of gestational diabetes, aRR = 1.28 (95% CI 1.01–1.62) [30]. Although additional monitoring is indicated, due to the absence of teratogenic concerns and its demonstrated broad activity in treating and preventing various phases of bipolar disorder, quetiapine should be considered a first-line alternative to valproate in those where either acute or ongoing maintenance treatment is indicated.

### Risperidone

Risperidone has a demonstrated evidence base in the management of bipolar disorder, primarily in managing acute manic and mixed episodes [6–9]. CANMAT guidelines recommend risperidone as a first-line treatment in acute mania and, specifically the long-acting injectable form as a second-line option for maintenance therapy [6, 7]. According to NICE, risperidone should be considered in managing mania or hypomania in individuals not already prescribed psychotropic treatment, or in addition to lithium. NICE also endorses risperidone for long-term management of bipolar disorder when lithium is ineffective or unsuitable [9]. WFSBP guidance highlights risperidone's efficacy primarily in treating acute and preventing relapse of manic episodes. [10, 12].

In *utero* exposure to risperidone is not anticipated to increase risk of congenital abnormalities. Neonatal complications, labelled as withdrawal symptoms have been identified following third trimester neonatal exposure [31]. These include extrapyramidal side effects, sedation, breathing and feeding difficulties, agitation and increased or decreased muscle tone. Some studies have reported an increased risk of gestational diabetes associated with risperidone use, although this has not been consistently demonstrated [31, 32]. Given the availability of alternative mood stabilising medications, including second-generation antipsychotics with a more robust evidence-base particularly in preventing relapse of manic episodes, risperidone should be reserved as an alternative to valproate to be considered after other options, including quetiapine and olanzapine, have been demonstrated as ineffective.

## Discussion

Within this narrative review, we have outlined mood stabilising medications that should be considered as both first- and second-line alternatives to valproate among individuals of child-bearing potential, particularly those planning a pregnancy. With new restrictions on the use of valproate in individuals of child-bearing age, options for suitable mood stabilising medications in bipolar disorder have now reduced. Choosing an alternative mood stabilising medication is challenging for clinicians and patients. Bipolar disorder is a heterogenous illness; different mood stabilising medications are associated with varying degrees of efficacy in both managing and preventing bipolar depression, mania, mixed mood presentations and rapid cycling. Furthermore, pregnancy is a vulnerable period for the mental health of pregnant people and often inadequately considered is the risk to the foetus from untreated, or inadequately treated, maternal mental illness [21]. Thus, whilst consideration of teratogenicity and risks in pregnancy of alternative mood stabilising medications is paramount, empirical evidence supporting the efficacy of alternative options much also be given due consideration.

Quetiapine can be considered the most well-rounded mood stabiliser in the management of BPAD, given its documented efficacy in treating and preventing episodes of mania, depression and mixed affective presentations [14, 16, 24, 28]. Quetiapine is not expected to increase the risk of congenital abnormalities based on available evidence [29], although in comparison to valproate, alternative concerns include an increased risk of clinically significant weight gain and gestational diabetes [29]. Considered together, and notwithstanding the need for monitoring, quetiapine should be considered one of the first-line alternatives to valproate in individuals of child-bearing age. Particularly during active pregnancy planning, aripiprazole and olanzapine can be considered first-line alternatives, where quetiapine is not suitable.

A 2025 umbrella review identified in utero lithium exposure to be associated with an increased risk of cardiac and congenital malformations following first trimester exposure, (estimated odds ratio (OR) = 1.88 [95% confidence interval 1.26–2.81] and 1.97 [1.38–2.79], respectively) or any trimester exposure (estimated OR = 1.91 [1.01–3.63]) [17, 18]. Physiological changes during pregnancy also alter lithium’s pharmacokinetic parameters necessitating increasing monitoring and dose adjustments to account for its narrow therapeutic index [20]. Thus, although the documented absolute risk increases are low [21], in those where second-generation antipsychotics are a viable option, these should be given preference. Although lamotrigine offers a safer alternative in pregnancy, it’s spectrum of activity in effectively managing bipolar disorder contrasts significant with that of valproate [22, 24]. It’s lack of efficacy in preventing manic episodes compared to lithium or second-generation antipsychotics limit its clinical utility as an alternative to those where valproate treatment is clinically indicated as an antimanic agent [22]. Carbamazepine should be avoided in people planning pregnancy due to concerns for teratogenicity [15].

Additional considerations informing choice of alternative mood stabiliser not discussed here should also inform treatment recommendations, including a person’s preferences and their physical and mental illness history. Thus, the information contained here is a guide for clinicians as to what medications should be prioritised for initial consideration, rather than definitive treatment recommendations. The management of bipolar disorder is complex, and many individuals require combination mood stabiliser treatment. Thus, the recommendations contained here need to be considered in that context. Whilst primary toxicity concerns are addressed here, this review does not contain a definitive review of all risks associated with in utero exposure to each considered mood stabilising medication, rather, those of primary concern, again to inform prioritisation of suitable alternatives by clinicians. Finally, the information contained here is limited by available research. Some safety concerns relating to each medication were identified in treatment of epilepsy versus bipolar disorder. It is possible that risks and associated estimates of risk are different within different populations [18]. As in all clinical populations, deficits within research conducted among pregnant individuals, particularly it’s largely retrospective nature and the impact of confounding variables, limit the confidence in reported estimates and subsequently the strength of conclusions that can be drawn.

## Conclusion

This narrative review provides an overview of alternative mood stabilising medications to sodium valproate in the management of bipolar disorder among people of child-bearing potential. The comparative efficacy and safety in individuals of child-bearing age of alternative mood stabilising medications must be given due consideration by clinicians. Quetiapine should be considered as a first-line alternative to valproate in people of child-bearing potential, as it is supported by robust empirical evidence in treating and preventing manic, depressive and mixed mood presentations. In those planning a pregnancy, olanzapine and aripiprazole can be considered as second-line alternatives where treatment with a second-generation antipsychotic is appropriate. Although the documented absolute risk increases are low, lithium use during pregnancy has been associated with an increased risk of congenital -particularly cardiac—malformations. Altered drug handling due to physiological changes of pregnancy can also complicate safe use. Among those not previously stabilised on lithium and planning a pregnancy, second-generation antipsychotics should be given preference. Although lamotrigine offers a safer alternative in pregnancy, it’s lack of efficacy in preventing manic episodes in particular limit its clinical utility as an alternative to those where valproate treatment is indicated. Carbamazepine treatment should be avoided due to similar concerns for teratogenicity.
